# Seizure Classification From EEG Signals Using an Online Selective Transfer TSK Fuzzy Classifier With Joint Distribution Adaption and Manifold Regularization

**DOI:** 10.3389/fnins.2020.00496

**Published:** 2020-06-11

**Authors:** Yuanpeng Zhang, Ziyuan Zhou, Heming Bai, Wei Liu, Li Wang

**Affiliations:** ^1^Department of Medical Informatics of Medical (Nursing) school, Nantong University, Nantong, China; ^2^Research Center for Intelligence Information Technology, Nantong University, Nantong, China

**Keywords:** seizure classification, brain-computer interface, transfer learning, joint distribution adaption, manifold regularization, TSK fuzzy classifier

## Abstract

To recognize abnormal electroencephalogram (EEG) signals for epileptics, in this study, we proposed an online selective transfer TSK fuzzy classifier underlying joint distribution adaption and manifold regularization. Compared with most of the existing transfer classifiers, our classifier has its own characteristics: (1) the labeled EEG epochs from the source domain cannot accurately represent the primary EEG epochs in the target domain. Our classifier can make use of very few calibration data in the target domain to induce the target predictive function. (2) A joint distribution adaption is used to minimize the marginal distribution distance and the conditional distribution distance between the source domain and the target domain. (3) Clustering techniques are used to select source domains so that the computational complexity of our classifier is reduced. We construct six transfer scenarios based on the original EEG signals provided by the Bonn University to verify the performance of our classifier and introduce four baselines and a transfer support vector machine (SVM) for benchmarking studies. Experimental results indicate that our classifier wins the best performance and is not very sensitive to its parameters.

## Introduction

**Figure 1:**
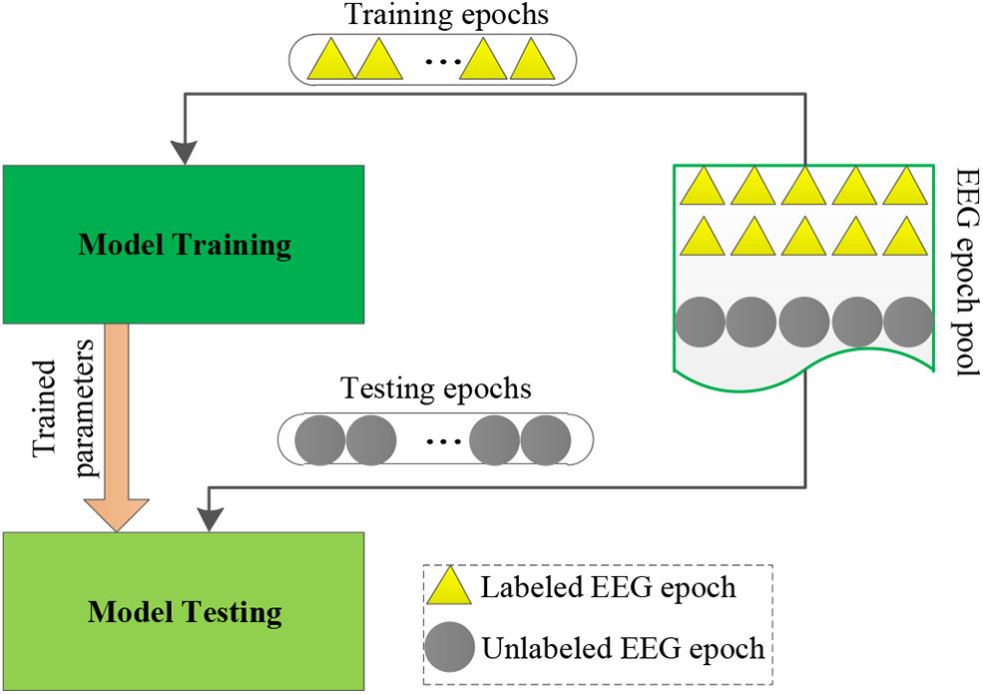
The classic diagnostic procedure for epilepsy.

The maturity of the brain–computer interface (BCI) technology has provided an important channel for the human to use artificial intelligence (AI) to explore the cognitive activities of the brain. For example, many AI methods have been proposed for an intelligent diagnosis of epilepsy instead of neurological physicians through electroencephalogram (EEG) signals (Ghosh-Dastidar et al., 2008; Van Hese et al., 2009; Wang et al., 2016). In this study, we also focus on the intelligent diagnosis of epilepsy through EEG signals. The classic diagnostic procedure for epilepsy by using intelligent models is illustrated in Figure 1. We observe that, for an emerging task, a large number of labeled EEG epochs are required to train an intelligent model. Therefore, it needs to consume a lot of effort to manually label EEG epochs. Because the responses to EEG signals of different patients in the same cognitive activity show a certain degree of similarity, we expect to leverage abundant labeled EEG epochs, which are available in a related source domain for training an accurate intelligent model to be reused in the target domain. To this end, transfer learning is often used, which has been proven to be promising for epilepsy EEG signal recognition. For example, Yang et al. (2014) proposed a transfer model LMPROJ for epilepsy EEG signal recognition underlying the support vector machine (SVM) framework. In LMPROJ, the marginal probability distribution distance measured by the maximal mean discrepancy (MMD) between the source domain and the target domain is used to minimize the distribution difference. Jiang et al. (2017c) improved LMPROJ and generated a model A-TL-SSL-TSK for epilepsy EEG signal recognition underlying the TSK fuzzy system framework. Comparing with LMPROJ, A-TL-SSL-TSK not only used the marginal probability distribution consensus as a transfer principle but also introduced semisupervised learning (cluster assumption) for regularization. Additionally, in our previous work (Jiang et al., 2020), we proposed an online multiview and transfer model O-MV-T-TSK-FS for EEG-based drivers' drowsiness estimation. It minimized not only the marginal distribution differences but also the conditional distribution differences between the source domain and the target domain. But it did not derive any information from unlabeled data. More references about transfer learning for epilepsy EEG signal recognition can be found in Jiang et al. (2019) and Parvez and Paul (2016).

Although existing intelligent models, for example, LMPROJ and A-TL-SSL-TSK, underlying the transfer learning framework are effective for epilepsy EEG signal recognition, there still exist some issues that should be further addressed.

- To tolerate the distribution difference between the source domain and the target domain, it is not enough to only minimize the marginal distribution difference between the two domains.
- Most of the existing models use only one source domain for knowledge transfer. That is to say, all available labeled data in the source domain are leveraged for model training. However, some labeled data may cause negative transfer.

Therefore, in this study, by overall considering the above two issues, we propose a new intelligent TSK fuzzy classifier (online selective transfer TSK fuzzy classifier with joint distribution adaption and manifold regularization, OS-JDA-MR-T-TSK-FC) for epilepsy EEG signal recognition. First, it further explores the marginal probability distribution adaption between the source domain and the target domain from two aspects. One is that it additionally introduces conditional probability distribution adaption to further minimize the distribution difference. The second is that it preserves manifold consistency underlying the marginal probability distribution. Second, it can selectively leverage knowledge from multiple source domains.

The following sections are organized as follows: in *Data and Methods*, we give the EEG data and our proposed method. In *Results*, we report the experimental results. Discussions about experimental results are presented in *Discussions*, and the whole conclusions are summarized in the last section.

## Data and Methods

### Data

**Figure 2:**
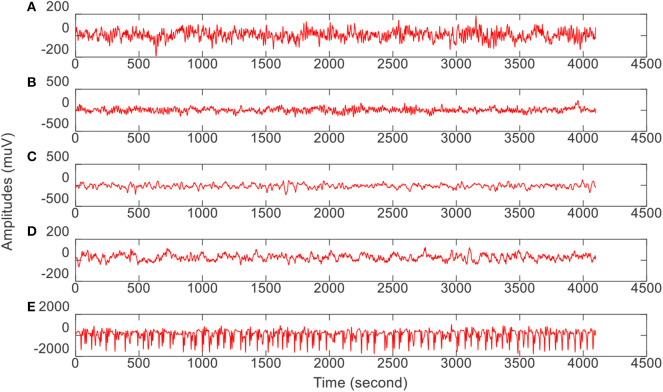
The amplitude of one volunteer in each group during the collection procedure. From top to bottom corresponds to **(A–E)**, respectively.

In this study, we download very commonly used epilepsy EEG^1^ data to verify our proposed intelligence model. The data from the University of Bonn is open to the public for scientific research. Table 1 gives the data archive and collection conditions. Additionally, Figure 2 illustrates the amplitudes during the collection procedure of one volunteer in each group. The original EEG data cannot be directly used for model training (Jiang et al., 2017b; Tian et al., 2019). We should employ feature extraction methods to extract robust features before model training.

**Table 1:** Epilepsy EEG data archive and collection condition.

**Volunteers**	**Groups**	**#Group**	**Collection conditions**
Health	A	100	Volunteers with eyes open
	B	100	Volunteers with eyes closed
Epileptic	C	100	From hippocampal formation during seizure free intervals
	D	100	From within epileptogenic zone during seizure free intervals
	E	100	During seizure activity

*Sampling rate: 173.6 Hz; duration: 23.6 s*.

#### Feature Extraction

Three feature extraction algorithms, that is, wavelet packet decomposition (WPD) (Li, 2011), short-time Fourier transform (STFT) (Pei et al., 1999), and kernel principal component analysis (KPCA) (Li et al., 2005), are employed to extract three kinds of features from the original epilepsy EEG signals.

- Wavelet Packet Decomposition

**Figure 3:**
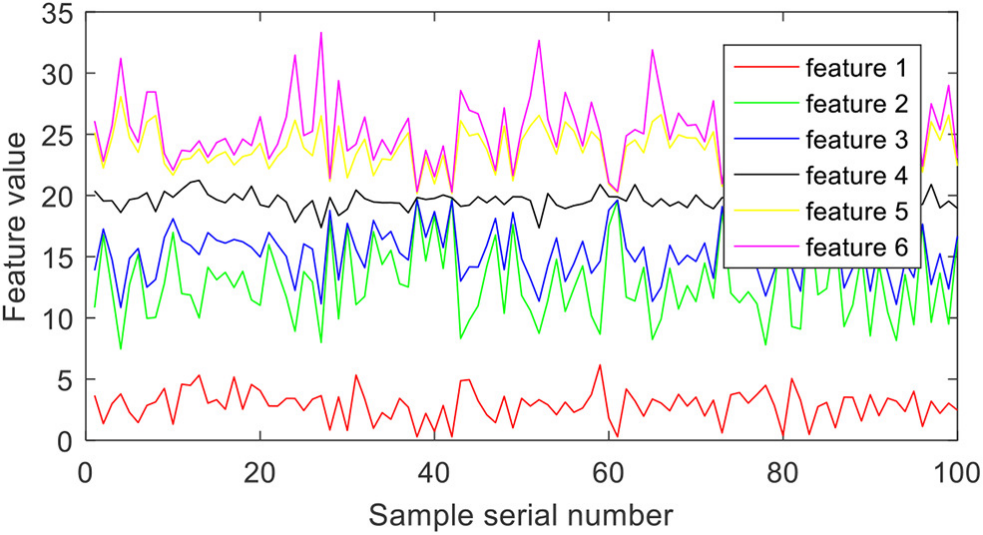
Features extracted by wavelet packet decomposition.

Wavelet packet decomposition is used to extract time-frequency features from epilepsy EEG signals. More specifically, the epilepsy EEG signals are disassembled into six different frequency bands with the Daubechies 4 wavelet coefficients. Each band is considered as one feature. Figure 3 illustrates the six features of group A.

- Short-Time Fourier Transform

**Figure 4:**
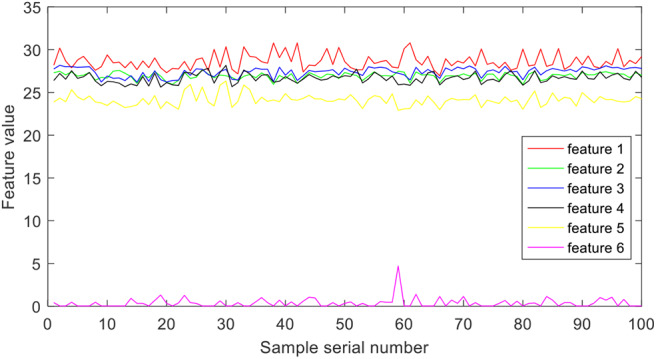
Features extracted by short time Fourier transform.

Short-time Fourier transform is used to extract frequency-domain features from epilepsy EEG signals. More specifically, the epilepsy EEG signals are disassembled into different local stationary signal segments, and then the Fourier transform is used to extract a group of spectra of the local segments, which are with evident time-varying characteristics at different times. Finally, six frequency bands are extracted from each group of spectra. Figure 4 illustrates the six features of group A.

- Kernel Principal Component Analysis

**Figure 5:**
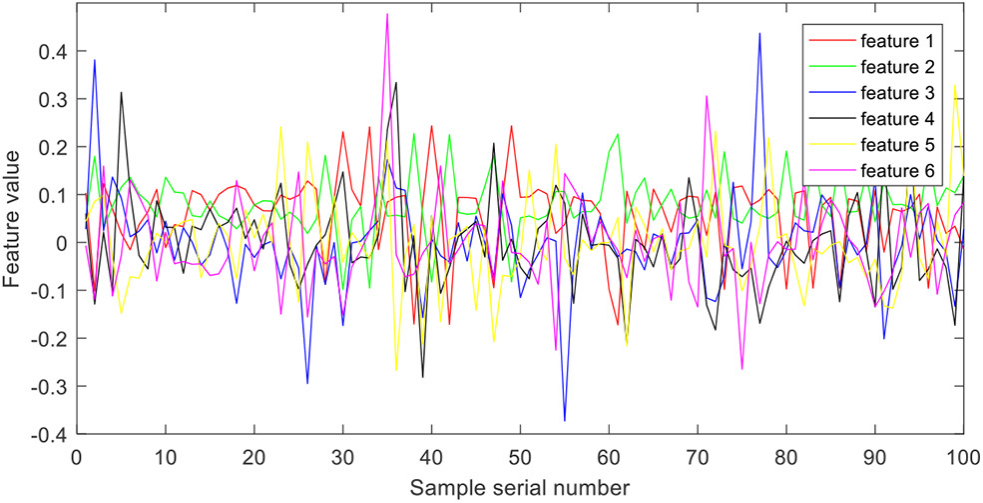
Features extracted by kernel principal component analysis.

Kernel principal component analysis is used to extract time-domain features from epilepsy EEG signals. More specifically, the Gaussian function is chosen as the kernel to map the original features nonlinearly. Then six kinds of features are selected from the top six PC eigenvectors. Figure 5 illustrates the six features of group A.

#### Online Transfer Scenario Construction

We construct six online transfer scenarios from the EEG data after feature extraction (Table 2). Each scenario consists of five source domains as multiple source domains and one target domain. Specifically, two healthy groups (A, B) and three epileptic groups (C, D, E) are combined to generate six different pairs of combinations, that is, AC, AD, AE, BC, BD, and BE. Five pairs are alternatively selected from the six combinations as source domains, and the rest one is taken as the target domain such that each pair has the opportunity to become the target domain.

**Table 2:** Six online transfer scenarios.

**Scenarios**	**Source domains**	**Target domain**	**No. of subject-specific objects**
SC-1	BD, BC, AE, AD, AC	BE	20
SC-2	BE, BC, AE, AD, AC	BD	20
SC-3	BE, BD, AE, AD, AC	BC	20
SC-4	BE, BD, BC, AD, AC	AE	20
SC-5	BE, BD, BC, AE, AC	AD	20
SC-6	BE, BD, BC, AE, AD	AC	20

**Figure 6:**
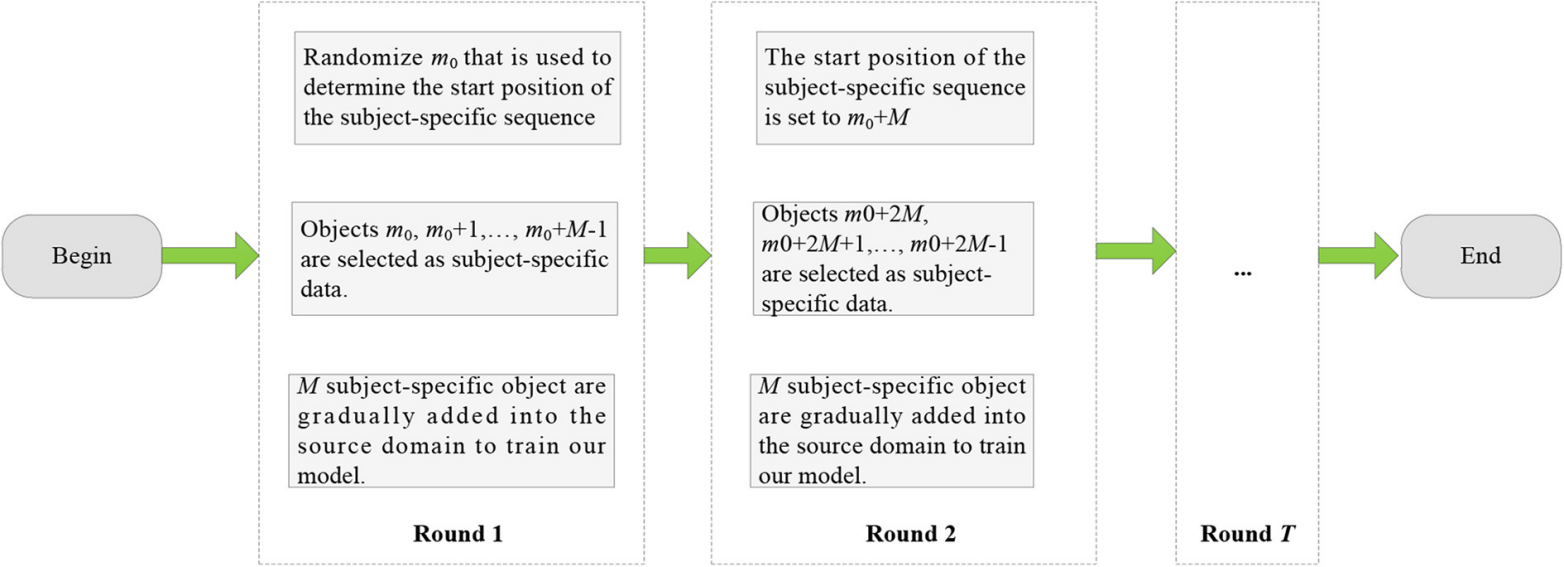
Online calibration flowchart.

In general, calibration in BCIs can be divided into two types, that is, offline calibration and online calibration (Jiang et al., 2020). Offline calibration means that we have obtained a pool of unlabeled EEG epochs. Some of unlabeled EEG epochs were labeled by experts to train a classifier. The unseen epochs then were classified by the trained classifier. Online calibration means that the training EEG epochs were obtained on-the-fly. That is to say, the classifier was trained online. Both calibration methods have their own advantages and disadvantages. For example, in offline calibration, unlabeled EEG epochs can be used to assist labeled ones to achieve classifier training, for example, semisupervised learning (Mallapragada et al., 2009; Zhang et al., 2013; Dornaika and El Traboulsi, 2016). Additionally, if necessary, we can easily obtain the label of any EEG epochs at any time. In online calibration, we not only have no unlabeled EEG epochs to be used for classifier training but also have little control on which epochs to see next. However, online calibration is more attractive because it is more in line with the needs of practical application scenarios. Therefore, in this study, we only consider online calibration for seizure classification. To simulate online calibration in the aforementioned six transfer scenarios, we first generate *M* = 20 subject-specific objects from the target domain. The online calibration flowchart is shown in Figure 6. We repeat all rounds 10 times to obtain statistically meaningful results, where each time has a random starting position *m*_0_.

### Methods

In this section, we will elaborate the method we proposed for seizure classification. We first mathematically state the transfer problem, and then we give the online transfer learning framework and hence the online transfer TSK fuzzy classifier (OS-JDA-MR-T-TSK-FC). Lastly, we give the detailed algorithm steps of OS-JDA-MR-T-TSK-FC including how to select source domains.

#### Problem Statement

A domain Ψ = {*X, P*(**x**)} in the transfer learning or domain adaption scenario consists of a *d*-dimensional feature space ∈ *R*^*d*^ and a marginal distribution *P*(**x**), and a task Γ = {*Y, P*(*y*|**x**)} in the similar scenario consists of a one-dimensional label space *Y* and a conditional distribution *P*(*y*|**x**), where *y* ∈ *Y*. Suppose that Ψ_*s*_ and Ψ_*t*_ are two domains derived from Ψ, they are deemed to be different when *X*_*s*_ ≠ *X*_*t*_ and/or *P*_*s*_(**x**) ≠ *P*_*t*_(**x**). Homoplastically, two tasks Γ_*s*_ and Γ_*t*_ derived from Γ are different when *Y*_*s*_ ≠ *Y*_*t*_ and/or *P*_*s*_(*y*|**x**) ≠ *P*_*t*_(*y*|**x**).

Based on the above definitions, the target of OS-JDA-MR-T-TSK-FC is to train a predictive function on a source domain Ψ_*s*_ having *N*-labeled EEG epochs ${\left\{\left({\text{x}}_{\text{i}},y_{i}\right)\right\}}_{i=1}^{N}$ and a target domain Ψ_*t*_ having *M*-labeled EEG subject-specific epochs ${\left\{\left({\text{x}}_{\text{i}},y_{i}\right)\right\}}_{i=1}^{M}$ to predict the class label of a unseen epoch in the target domain with a low expected error under the hypotheses that Ψ_*s*_ = Ψ_*t*_, *Y*_*s*_ = *Y*_*t*_, *P*_*s*_(**x**) ≠ *P*_*t*_(**x**), and *P*_*s*_(*y*|**x**) ≠ *P*_*t*_(*y*|**x**).

#### OS-JDA-MR-T-TSK-FC

- Online Transfer Learning Framework

Because the classic one-order TSK fuzzy classifier (1-TSK-FC) (Deng et al., 2015; Jiang Y. et al., 2017a; Zhang J. et al., 2018; Zhang et al., 2019) is considered as the basic component of our online transfer learning framework, we first give some details about 1-TSK-FC before introducing our framework.

The *k*th fuzzy rule involved in 1-TSK-FC is formulated as the following *if–then* form:

(1) $$\begin{array}{l} \text{If }x_{i1}\;\text{is }A_{1}^{k}\;\wedge x_{i2}\;\text{is }A_{2}^{k}\wedge \ldots \wedge x_{id}\;\text{is }A_{d}^{k},\\ \;\text{then }f^{k}\left({\text{x}}_{\text{i}}\right)=p_{0}^{k}+p_{1}^{k}x_{i1}+\ldots +p_{d}^{k}x_{id}, \end{array}$$

where *k* = 1, 2, …, *K*, *K* represents the total number of fuzzy rules 1-TSK-FC uses. ${\text{x}}_{\text{i}}={\left[x_{i1},x_{i2},\ldots ,x_{id}\right]}^{T}$ represents the *i*th object contains *d* features. $A_{j}^{k}$ in (1) represents a fuzzy set subscribed by *x*_*ij*_ for the *k*th fuzzy rule, and ∧ represents a fuzzy conjunction operator. Each fuzzy rule is premised on the feature space and maps the fuzzy sets in the feature space into a varying singleton represented by $f^{k}\left({\text{x}}_{\text{i}}\right)$. After the steps of inference and defuzzification, the predictive function *y*^*o*^(•) for an unseen object **x** is formulated as the following form:

(2) $$\begin{array}{l} y^{o}\left(\text{x}\right)=\overset{K}{\underset{k=1}{\sum }}\left(\mu^{k}\left(\text{x}\right)/\overset{K}{\underset{k^{\prime }=1}{\sum }}\mu^{k^{\prime }}\left(\text{x}\right)\right)f^{k}\left(\text{x}\right)=\overset{K}{\underset{k=1}{\sum }}\left(\tilde{\mu }\left(\text{x}\right)\right)f^{k}\left(\text{x}\right), \end{array}$$

in which the μ^*k*^(**x**) is expressed as

(3) $$\begin{array}{l} \mu^{k}\left(\text{x}\right)=\prod_{j=1}^{d}\mu_{A_{j}^{k}}\left(x_{j}\right), \end{array}$$

where $\mu_{A_{j}^{k}}\left(x_{j}\right)$ can be expressed as the following form when the Gaussian kernel function is employed:

(4) $$\begin{array}{l} \mu_{A_{j}^{k}}\left(x_{j}\right)=exp\left(-{\left(x_{j}-c_{j}^{k}\right)}^{2}/2{\left(\delta_{j}^{k}\right)}^{2}\right), \end{array}$$

where $c_{j}^{k}$ and $\delta_{j}^{k}$ are two parameters representing the kernel center and kernel width, respectively. Therefore, training of 1-TSK-FC means to find optimal $c_{j}^{k}$, $\delta_{j}^{k}$ in the *if* parts, and ${\text{p}}^{k}={\left[p_{0}^{k},p_{1}^{k},\ldots ,p_{d}^{k}\right]}^{T}$ in the *then* parts. Referring to the literature (Zhang et al., 2019), we know that parameters in the *if* parts can be trained by clustering techniques. For instance, $c_{j}^{k}$ and $\delta_{j}^{k}$ can be trained by fuzzy *c*-means (FCM) (Gu et al., 2017) as

(5) $$\begin{array}{l} c_{j}^{k}=\overset{N}{\underset{i=1}{\sum }}\mu_{ik}x_{ij}/\overset{N}{\underset{i=1}{\sum }}\mu_{ik} \end{array}$$

(6) $$\begin{array}{l} \delta_{j}^{k}=h\overset{N}{\underset{i=1}{\sum }}{\mu_{ik}\left(x_{ij}-c_{j}^{k}\right)}^{2}\overset{N}{\underset{i=1}{\sum }}\mu_{ik}, \end{array}$$

where μ_*ik*_ is the fuzzy membership degree of **x**_*i*_ belonging to the *k*th cluster. *h* is a regularized parameter that can be always set to 0.5 according to the suggestions in Jiang Y. et al. (2017a). When $c_{j}^{k}$ and $\delta_{j}^{k}$ in the *if* parts are determined by FCM or other similar techniques, for an object **x**_*i*_ in the training set, let

(7.a) $$\begin{array}{l} {\text{x}}_{e}={\left(1,{\left({\text{x}}_{\text{i}}\right)}^{T}\right)}^{T}, \end{array}$$

(7.b) $$\begin{array}{l} {\tilde{\text{x}}}_{i}^{k}={\tilde{\mu }}^{k}\left({\text{x}}_{i}\right){\text{x}}_{e}, \end{array}$$

(7.c) $$\begin{array}{l} {\text{x}}_{gi}={\left({\left({\tilde{\text{x}}}_{i}^{1}\right)}^{T},{\left({\tilde{\text{x}}}_{i}^{2}\right)}^{T},\ldots ,{\left({\tilde{\text{x}}}_{i}^{K}\right)}^{T}\right)}^{T}, \end{array}$$

(7.d) $$\begin{array}{l} {\text{p}}^{k}={\left(p_{0}^{k},p_{1}^{k},\ldots ,p_{d}^{k}\right)}^{T}, \end{array}$$

(7.e) $$\begin{array}{l} {\text{p}}_{g}={\left({\left({\text{p}}^{1}\right)}^{T},{\left({\text{p}}^{2}\right)}^{T},\ldots ,{\left({\text{p}}^{K}\right)}^{T}\right)}^{T}, \end{array}$$

then we can rewrite the predictive function *y*^*o*^(·) in (2) as the following form:

(8) $$\begin{array}{l} y^{o}\left({\text{x}}_{i}\right)={\text{p}}_{\text{g}}^{T}{\text{x}}_{gi} \end{array}$$

Referring to Zhou et al. (2017) and Zhang Y. et al. (2018), we formulate an objective function as follows to solve **p**_*g*_:

(9) $$\begin{array}{l} J_{1-order-TSK-Fc}\left({\text{p}}_{g}\right)=\frac{1}{2}{\left({\text{p}}_{g,c}\right)}^{T}{\text{p}}_{g,c}+\frac{\eta }{2}\overset{N}{\underset{i=1}{\sum }}{\left\|{\left({\text{p}}_{g}\right)}^{T}{\text{x}}_{gi}-y_{i}\right\|}^{2}, \end{array}$$

where the first $\frac{1}{2}{\left({\text{p}}_{g}\right)}^{T}{\text{p}}_{g}$ is a generalization term, the second is a square error term, and η > 0 is balance parameter used to control the tolerance of errors and the complexity of 1-TSK-FC. By setting the partial derivative of the objective function w.r.t **p**_*g*_ to zero, that is, ∂*J*_1−*order*−*TSK*−*FS*_(**p**_*g*_)/∂**p**_*g*_ = 0, we can compute **p**_*g*_ analytically as

(10) $$\begin{array}{l} {\text{p}}_{g}={\left({\text{I}}_{k\left(d+1\right)\times k\left(d+1\right)}+\overset{N}{\underset{i=1}{\sum }}{\text{x}}_{gi}{\left({\text{x}}_{gi}\right)}^{T}\right)}^{-1}\times \left(\eta \overset{N}{\underset{i=1}{\sum }}{\text{x}}_{gi}y_{i}\right). \end{array}$$

In this study, 1-TSK-FC is taken as the basic learning component to support the transfer learning framework. Many previous works (Yang et al., 2014; Jiang et al., 2017c) explored the marginal distribution adaption between the source domain and the target domain for transfer learning. In our framework, we introduce conditional distribution adaption to further minimize the distribution difference. Additionally, we impose manifold consistency on the marginal distribution. Therefore, the transfer learning framework can be formulated as

(11) $$\begin{array}{l} f=\arg\underset{f}{\min}\left[\overset{N}{\underset{i=1}{\sum }}\ell \left(f\left({\text{x}}_{i}\right),y_{i}\right)+\omega_{t}\overset{N+M}{\underset{i=N+1}{\sum }}\ell \left(f\left({\text{x}}_{i}\right),y_{i}\right)\right]\\ \;+\lambda_{1}\left[D\left(J_{s},J_{t}\right)\right]+\lambda_{2}\left[M\left(P_{s},P_{t}\right)\right], \end{array}$$

where ω_*t*_ in the first term is the overall weights of the specific-subject objects. Generally, ω_*t*_ should be larger than 1 so that more emphasis is given to objects in Ψ_*s*_ than Ψ_*t*_. Therefore, we set ω_*t*_ to ω_*t*_ = max(2, σ · *N*/*M*). λ_1_ and λ_2_ are regularization parameters. The first term contains two parts: the first is to measure the loss on Ψ_*s*_, and the second is to measure the loss in Ψ_*t*_. The second one is the joint distribution adaption term, and the third one is the manifold regularization term. Below, we will explain how to embody them formally.

- Objective function of OS-JDA-MR-T-TSK-FC

Under the framework shown in (11), we specify each term to get the objective function of our online transfer TSK fuzzy classifier OS-JDA-MR-T-TSK-FC.

#### Loss Function

The squared loss is taken as the loss function to measure the sum of squared training errors on both Ψ_*s*_ and Ψ_*t*_; hence, the first term in (11) can be formulated as

(12) $$\begin{array}{l} \overset{N}{\underset{i=1}{\sum }}{\left(f\left({\text{x}}_{i}\right)-y_{i}\right)}^{2}+\omega_{t}\overset{N+M}{\underset{i=N+1}{\sum }}{\left(f\left({\text{x}}_{i}\right)-y_{i}\right)}^{2}\\ \;=\overset{N}{\underset{i=1}{\sum }}{\left({\text{p}}_{g}^{T}{\text{x}}_{gi}-y_{i}\right)}^{2}+\omega_{t}\overset{N+M}{\underset{i=N+1}{\sum }}{\left({\text{p}}_{g}^{T}{\text{x}}_{gi}-y_{i}\right)}^{2}, \end{array}$$

where $f\left(\text{x}\right)={\text{p}}_{g}^{T}{\text{x}}_{gi}$ is the predictive function of 1-TSK-FC. Suppose we have a diagonal matrix Θ in which each element is defined as

(13) $$\begin{array}{l} \Theta \left(i,i\right)=\{\begin{array}{ll} 1 & 1\leq i\leq N\\ \omega_{t} & N+1\leq i\leq N+M \end{array}. \end{array}$$

By submitting (13) to (12), then (12) can be rewritten as

(14) $$\begin{array}{l} \overset{N}{\underset{i=1}{\sum }}{\left({\text{p}}_{g}^{T}{\text{x}}_{gi}-y_{i}\right)}^{2}+\omega_{t}\overset{N+M}{\underset{i=N+1}{\sum }}{\left({\text{p}}_{g}^{T}{\text{x}}_{gi}-y_{i}\right)}^{2}\\ \;=\overset{N+M}{\underset{i=1}{\sum }}\Theta \left(i,i\right){\left({\text{p}}_{g}^{T}{\text{x}}_{gi}-y_{i}\right)}^{2}\\ \;=\left({\text{y}}^{T}-{\text{p}}_{g}^{T}{\text{X}}_{g}^{T}\right)\Theta \left(\text{y}-{\text{X}}_{g}{\text{p}}_{g}\right), \end{array}$$

where ${\text{X}}_{g}={\left[{\text{X}}_{g1},\ldots ,{\text{X}}_{gN},\ldots ,{\text{X}}_{g\left(N+M\right)}\right]}^{T}$ in which each element ${\text{X}}_{gi}$ is derived from ${\text{X}}_{i}$ by using (7.c).

##### Joint distribution adaptation

As all we know that even EEG epoch features in Ψ_*s*_ and Ψ_*t*_ are extracted in the same way, the joint distributions (marginal and conditional distributions) between Ψ_*s*_ and Ψ_*t*_ are generally different. In order to meet practical requirements, we assume that *P*_*s*_(**x**) ≠ *P*_*t*_(**x**) and *P*_*s*_(*y*|**x**) ≠ *P*_*t*_(*y*|**x**). Therefore, a joint distribution adaptation should be designed to minimize the distribution similarity (distance) *D*(*J*_*s*_, *J*_*t*_) between Ψ_*s*_ and Ψ_*t*_.

First, the projected MMD (Gangeh et al., 2016; Jia et al., 2018; Lin et al., 2018) is employed to the marginal distribution similarity *D*(*P*_*s*_, *P*_*t*_) between Ψ_*s*_ and Ψ_*t*_. As a result, *D*(*P*_*s*_, *P*_*t*_) can be expressed as

(15) $$\begin{array}{l} D\left(P_{t},P_{s}\right)={\left[\frac{1}{N}\overset{N}{\underset{i=1}{\sum }}f\left({\text{x}}_{i}\right)-\frac{1}{M}\overset{N+M}{\underset{i=N+1}{\sum }}f\left({\text{x}}_{i}\right)\right]}^{2}={\text{p}}_{g}^{T}{\text{X}}_{g}\Phi {\text{X}}_{g}{\text{p}}_{g}, \end{array}$$

where Φ is the MMD matrix, which can be defined as

(16) $$\begin{array}{l} \Phi \left(i,j\right)=\{\begin{array}{ll} 1/N^{2},\; & 1\leq i\leq N,\text{1}\leq j\leq N\\ 1/M^{2},\; & N+\text{1}\leq i,j\leq N+M\\ -1/NM\; & \text{otherwise}. \end{array} \end{array}$$

Second, we suppose that Ψ_*s,c*_ belongs to Ψ_*s*_ and its objects are selected by {**x**_**i**_**|x**_**i**_ ∈ Ψ_*s*_ ∧ *y*_*i*_ = *c*}, and Ψ_*t,c*_ belongs to Ψ_*t*_ and its objects are selected by {**x**_**i**_**|x**_**i**_ ∈ Ψ_*t*_ ∧ *y*_*i*_ = *c*}, where *c* means the *c*th class in one domain. Also, for the source domain, *N*_*c*_ is used to denote the number of objects in the *c*th class, and for the specific-subject objects in the target domain, *M*_*c*_ is used to denote the number of objects in the *c*th class. Hence, *D*(*Q*_*s*_, *Q*_*t*_) can be expressed as

(17) $$\begin{array}{l} D\left(Q_{t},Q_{s}\right)=\overset{2}{\underset{c=1}{\sum }}{\left[\frac{1}{N_{c}}\underset{{\text{x}}_{i}\in \Omega_{s,c}}{\sum }f\left({\text{x}}_{i}\right)-\frac{1}{M_{c}}\underset{{\text{x}}_{j}\in \Omega_{t,c}}{\sum }f\left({\text{x}}_{j}\right)\right]}^{2}\\ \;=\overset{2}{\underset{c=1}{\sum }}{\left[\frac{1}{N_{c}}\underset{{\text{x}}_{i}\in \Omega_{s,c}}{\sum }{\text{p}}_{g}^{T}{\text{x}}_{gi}-\frac{1}{M_{c}}\underset{{\text{x}}_{j}\in \Omega_{t,c}}{\sum }{\text{p}}_{g}^{T}{\text{x}}_{gj}\right]}^{2},\\ \;=\overset{2}{\underset{c=1}{\sum }}{\text{p}}_{g}^{T}{\text{X}}_{g}\Delta_{c}{\text{X}}_{g}{\text{p}}_{g},\\ \;={\text{p}}_{g}^{T}{\text{X}}_{g}\Delta {\text{X}}_{g}{\text{p}}_{g}, \end{array}$$

where $\Delta =\overset{2}{\underset{c=1}{\sum }}\Delta_{c}$ and Δ_*c*_ is an MMD matrix defined as follows:

(18) $$\begin{array}{l} \Delta_{c}\left(i,j\right)=\{\begin{array}{ll} 1/N_{c}^{2}\; & {\text{x}}_{i},{\text{x}}_{j}\in \Omega_{s,c}\\ 1/M_{c}^{2}\; & {\text{x}}_{i},{\text{x}}_{j}\in \Omega_{t,c}\\ -1/N_{c}M_{c}\; & {\text{x}}_{i}\in \Omega_{s,c},{\text{x}}_{j}\in \Omega_{t,c}\;\\ \; & \text{or }{\text{x}}_{i}\in \Omega_{t,c},{\text{x}}_{j}\in \Omega_{s,c}\\ 0\; & \text{otherwise} \end{array} \end{array}$$

According to the probability theory, the joint adaption *D*(*J*_*s*_, *J*_*t*_) = *D*(*P*_*s*_, *P*_*t*_)+*D*(*Q*_*s*_, *Q*_*t*_) so that the joint distribution adaptation can be formulated as

(19) $$\begin{array}{l} D\left(J_{s},J_{t}\right)=D\left(P_{t},P_{s}\right)+D\left(Q_{t},Q_{s}\right)\\ \;={\text{p}}_{g}^{T}{\text{X}}_{g}\Phi {\text{X}}_{g}{\text{p}}_{g}+{\text{p}}_{g}^{T}{\text{X}}_{g}\Delta {\text{X}}_{g}{\text{p}}_{g},\\ \;={\text{p}}_{g}^{T}{\text{X}}_{g}\left(\Phi +\Delta \right){\text{X}}_{g}{\text{p}}_{g}. \end{array}$$

##### Manifold regularization

In the manifold assumption (Lin and Zha, 2008; Chen and Wang, 2011; Geng et al., 2012), it is assumed that if two objects **x**_**i**_ and **x**_**j**_ are very close in the intrinsic geometry in terms of *P*(**x**_**i**_) and *P*(**x**_**j**_), then the corresponding *Q*(*y*_*i*_**|x_i_**) and *Q*(*y*_*j*_**|x**_*j*_) are considered as being similar. That is to say, for the objects in Ψ_*s*_ and the calibration objects in Ψ_*t*_, if they are in a manifold, it is expected that their output (conditional probability distribution) differences should be as small as possible. Therefore, the manifold regularization can be formulated as follows under geodesic smoothness,

(20) $$\begin{array}{l} M\left(P_{s},P_{t}\right)=\overset{N+M}{\underset{i=1}{\sum }}\overset{N+M}{\underset{j=1}{\sum }}{\left(f\left({\text{x}}_{i}\right)-f\left({\text{x}}_{j}\right)\right)}^{2}w_{ij}\\ \;=\overset{N+M}{\underset{i=1}{\sum }}\overset{N+M}{\underset{j=1}{\sum }}f\left({\text{x}}_{i}\right)l_{ij}f\left({\text{x}}_{j}\right)\\ \;=\overset{N+M}{\underset{i=1}{\sum }}\overset{N+M}{\underset{j=1}{\sum }}{\text{p}}_{g}^{T}{\text{x}}_{gi}l_{ij}{\text{p}}_{g}^{T}{\text{x}}_{gj}\\ \;={\text{p}}_{g}^{T}{\text{X}}_{g}\text{L}{\text{X}}_{g}{\text{p}}_{g}, \end{array}$$

Where, **W** = [*w*_*ij*_]_(*N*+*M*)×(*N*+*M*)_ is the graph affinity matrix in which each element is defined as

(21) $$\begin{array}{l} w_{ij}=\{\begin{array}{ll} \cos\left({\text{x}}_{i},{\text{x}}_{j}\right)\; & \text{if }{\text{x}}_{i}\in \xi_{\nu }\left({\text{x}}_{j}\right)\text{ or }{\text{x}}_{j}\in \xi_{\nu }\left({\text{x}}_{i}\right)\\ 0\; & \text{otherwise} \end{array}, \end{array}$$

Where, ξ_ν_(**x**_*i*_) represents a set of *v*-nearest neighbors of object **x**_*i*_. **L** = [*l*_*ij*_]_(*N*+*M*)×(*N*+*M*)_ is the corresponding normalized graph Laplacian matrix of **W**, which can be computed by **L** = **I** − **D**^−1/2^**WD**^−1/2^, where **D** is the degree matrix in which each diagonal element *d*_*ii*_ is computed by $\overset{N+M}{\underset{j=1}{\sum }}w_{ij}$.

By embedding the manifold regularization into the transfer learning framework, the marginal probability distributions of objects in the target domain and the source domain are fully utilized to guarantee the consistency between the predictive structure of the decision function *f* and the intrinsic manifold data structure.

By substituting (14), (19), and (20) into our transfer learning framework shown in (12), we can obtain a transfer learning model, that is, OS-JDA-MR-T-TSK-FC as

(22) $$\begin{array}{l} f=\arg\underset{f}{\min}[\left({\text{y}}^{T}-{\text{p}}_{g}^{T}{\text{X}}_{g}^{T}\right)\Theta \left(\text{y}-{\text{X}}_{g}{\text{p}}_{g}\right)\\ \;+{\text{p}}_{g}^{T}{\text{X}}_{g}\lambda_{1}\left(\Phi +\Delta \right){\text{X}}_{g}{\text{p}}_{g}+{\text{p}}_{g}^{T}{\text{X}}_{g}\lambda_{2}\text{L}{\text{X}}_{g}{\text{p}}_{g}],\\ \;=\arg\underset{f}{\min}[\left({\text{y}}^{T}-{\text{p}}_{g}^{T}{\text{X}}_{g}^{T}\right)\Theta \left(\text{y}-{\text{X}}_{g}{\text{p}}_{g}\right)\\ \;+{\text{p}}_{g}^{T}{\text{X}}_{g}\left(\lambda_{1}\left(\Phi +\Delta \right)+\lambda_{2}\text{L}\right){\text{X}}_{g}{\text{p}}_{g}]. \end{array}$$

We can deduce a closed-form solution of **p**_*g*_ for the objective function in (26) by setting its derivative w.r.t **p**_*g*_ to zero as

(23) $$\begin{array}{l} {\text{p}}_{g}={\left[{\text{X}}_{g}^{T}\left(\Theta +\lambda_{1}\Phi +\lambda_{1}\Delta +\lambda_{2}\text{L}\right){\text{X}}_{g}\right]}^{-1}{\text{X}}_{g}^{T}\Theta \text{y}. \end{array}$$

#### Algorithm of OS-JDA-MR-T-TSK-FC

Different from most of the existing transfer models, OS-JDA-MR-T-TSK-FC can leverage knowledge from multiple source domains. However, as we know, too many source domains will improve computational complexity. Additionally, some source domains having significant differences with the target domain may bring some negative transfer knowledge. Therefore, according to Wu et al. (2017), we adopt a distance-based schema to select relative source domains.

We use **v**_*z,c*_ to denote the mean vector of each class in the *z*th source domain, where *z* = 1,2,…, *Z*. Similarly, **v**_*t,c*_ is used to denote the mean vector of each class in the target domain. The Euclidean distance between the *z*th source domain and the target domain can be computed as

(24) $$\begin{array}{l} d\left(z,t\right)={\underset{c}{\sum }\|{\text{v}}_{z,c}-{\text{v}}_{t,c}\|}^{2}. \end{array}$$

With (24), we can get a distance set {*d*(1, *t*), *d*(2, *t*), …, *d*(*Z, t*)} that contains *Z* domain distances. The distance set then is partitioned by *k*-means to *k* groups (in this study, *k* is set to 2), and the source domains are selected from the cluster who has the smallest center.

As a whole, the training of OS-JDA-MR-T-TSK-FC contains three parts: the first one is source domain selection, the second one is model training on a source domain combining with the target domain, and the last is classifier combination. Algorithm 1 shows the detailed training steps of OS-JDA-MR-T-TSK-FC.

**Algorithm 1:** OS-JDA-MR-T-TSK-FC

**Input**: 1. ${\left[\left({\text{x}}_{1},y_{1}\right),\left({\text{x}}_{2},y_{2}\right),\ldots ,\left({\text{x}}_{N},y_{N}\right),\ldots ,\left({\text{x}}_{N+M},y_{N+M}\right)\right]}^{T}$ 2. ω_*t*_, λ_1_, λ_2_ and the number of fuzzy rules *K*; **Output**: 1. Training accuracy α_*z*_ of each classifier; 2. Final decision function *f*; **Procedure:** **For** *z* = 1 to *Z* Calculate the Euclidean distance *d*(*z, t*) between the *z*th source domain and the target domain by (24). **End** Partition the distance set {*d*(1, *t*), *d*(2, *t*), …, *d*(*Z, t*)} into two groups. Select *Z*/2 (as *Z′*) source domains from *Z* source domains. **For** *z* = 1 to *Z′* Map **X** to **X**_*g*_ by (7.c); Calculate **Θ**, **Φ**, **Δ**, and **L** by (13), (16), and (18), respectively. Calculate **p**_*g*_ and record it as (**p**_*g*_)_*z*_ by (23); Use (**p**_*g*_)_*z*_ to predict *N*_*z*_+*M* objects the record the training accuracy as α_*z*_; **End** Return $f\left(\text{x}\right)=\alpha_{1}{\left({\text{p}}_{g}^{T}\right)}_{1}{\text{x}}_{g}+\alpha_{2}{\left({\text{p}}_{g}^{T}\right)}_{2}{\text{x}}_{g}+\ldots +\alpha_{Z^{\prime }}{\left({\text{p}}_{g}^{T}\right)}_{Z^{\prime }}{\text{x}}_{g}$;

OS-JDA-MR-T-TSK-FC can also be used for multiclassification tasks. According to Zhou et al. (2017), we can convert **y** from the space *R* to the space *R*^*C*^ by that *y*_*ij*_ = 1 if *y*(**x**_*i*_) = *j*, and *y*_*ij*_ = 0 otherwise, where *i* = 1, 2, …, *N* + *M*, *j* = 1, 2, …, *C*, and *C* represents the number of classes. Thus, the label space becomes $\text{Y}={\left[{\text{y}}_{1},\ldots ,{\text{y}}_{N},\ldots ,{\text{y}}_{N+M}\right]}^{T}\in R^{C}$, and ${\text{p}}_{g}$ is also converted from *R*^*d*+1^ to *R*^(*d*+1)×*C*^.

## Results

Experiment setups and comparison results will be reported in this section.

### Setups

For fair, we introduce three baselines and one transfer learning algorithm for comparison study. The three baselines all use 1-TSK-FC for training. But their training sets are different.

1. Baseline 1 (BL1). Its training set consists of the five source domains directly connected, and its testing set is the target domain. Therefore, BL1 is considered as a calibration-independent classifier, which does not use the subject-specific data in the target domain for training.
2. Baseline 2 (BL2). It uses only subject-specific calibration EEG data in the target domain for training. Its testing set is the unlabeled data in the target domain. Therefore, BL2 is considered as a source domain-independent classifier, which does not consider the EEG data in the source domains at all.
3. Baseline 3 (BL3). BL3 is trained on five training sets, receptively. Each set consisted of a source domain and the subject-specific data in the target domain. The five trained models are finally combined by a weight schema that is also used in Algorithm 1. Its testing set is the unlabeled data in the target domain
4. Transfer support vector machine (TSVM) (Chapelle et al., 2008). It trains five TSVM classifiers by combining unlabeled EEG data in the target domain for semisupervised learning. The five trained models are finally combined by a weight schema that is also used in Algorithm 1.
5. ARRLS (Long et al., 2014). It trains five ARRLS classifiers by combining unlabeled EEG data in the target domain for supervised learning. The five trained models are finally combined by a weight schema that is also used in Algorithm 1.

### Experimental Results

In this section, we report the experimental results from several aspects, that is, classification performance, interpretability, and robustness.

- Classification Performance

Table 3 shows the average classification performance of the six scenarios in the KPCA feature space, PWD feature space, and STFT feature space, respectively. Table 4 shows the classification performance on KPCA features. Table 5 shows the classification performance on PWD features, and Table 6 shows the classification performance on STFT features. The best results are marked in bold.

- Interpretability

**Table 3:** Average classification performance of the six scenarios in three feature spaces.

	***M***	**0**	**4**	**8**	**12**	**16**	**20**
KPCA	BL1	0.7962	0.7962	0.7962	0.7962	0.7962	0.7962
	BL2	—	0.6837	0.7460	0.7899	0.8270	0.8536
	BL3	0.7881	0.7761	0.8016	0.8086	0.8048	0.8174
	TSVM	**0.8723**	0.8765	0.8810	0.8864	0.8811	0.8927
	ARRLS	0.8684	0.8217	0.8742	0.8684	0.8821	0.8823
	OS-JDA-MR-T-TSK-FC	0.8701	**0.8943**	**0.9164**	**0.9191**	**0.9214**	**0.9251**
PWD	BL1	0.8618	0.8618	0.8618	0.8618	0.8618	0.8618
	BL2	—	0.7151	0.8597	0.8867	0.9057	0.9176
	BL3	0.8505	0.8503	0.8661	0.8685	0.8751	0.8795
	TSVM	**0.9232**	**0.9271**	0.9269	0.9312	0.9292	0.9344
	ARRLS	0.9157	0.9204	0.9224	0.9287	0.9312	0.9336
	OS-JDA-MR-T-TSK-FC	0.8864	0.9073	**0.9278**	**0.9314**	**0.9332**	**0.9376**
STFT	BL1	0.9129	0.9129	0.9129	0.9129	0.9129	0.9129
	BL2	—	0.7619	0.8531	0.8674	0.8873	0.8962
	BL3	0.9011	0.8923	0.8924	0.8951	0.8989	0.9107
	TSVM	0.9365	**0.9459**	0.9467	0.9502	0.9581	0.9524
	ARRLS	**0.9425**	0.9410	0.9356	0.9478	0.9452	0.9550
	OS-JDA-MR-T-TSK-FC	0.9031	0.9214	**0.9500**	**0.9517**	**0.9585**	**0.9619**

*The best performance is marked in bold*.

**Table 4:** Classification performance on six scenarios in the KPCA feature space.

	***M***	**0**	**4**	**8**	**12**	**16**	**20**
SC-1	BL1	0.7254	0.7254	0.7253	0.7253	0.7253	0.7253
	BL2	—	0.6507	0.6949	0.7285	0.7438	0.8124
	BL3	0.7845	0.7899	0.8283	0.8535	0.8332	0.8404
	TSVM	0.8527	0.8564	0.8661	0.8675	0.8684	0.8690
	ARRLS	0.8455	0.8631	0.8874	0.8584	0.8632	0.8741
	OS-JDA-MR-T-TSK-FC	0.8835	0.9124	0.9187	0.9123	0.9201	0.9206
SC-2	BL1	0.8050	0.8050	0.8050	0.8050	0.8050	0.8050
	BL2	—	0.6031	0.7458	0.8727	0.9242	0.9447
	BL3	0.7811	0.7912	0.8821	0.8642	0.8097	0.8358
	TSVM	0.9231	0.9305	0.9289	0.9359	0.9399	0.9378
	OS-JDA-MR-T-TSK-FC	0.9187	0.9364	0.9397	0.9415	0.9434	0.9439
SC-3	BL1	0.9045	0.9045	0.9045	0.9045	0.9045	0.9045
	BL2	—	0.8079	0.8689	0.8667	0.8418	0.9191
	BL3	0.8008	0.7838	0.8037	0.8165	0.7804	0.8239
	TSVM	0.9235	0.9214	0.9298	0.9311	0.9287	0.9324
	ARRLS	0.9154	0.9200	0.9147	0.9228	0.9142	0.9364
	OS-JDA-MR-T-TSK-FC	0.9111	0.9125	0.9341	0.9399	0.9421	0.9433
SC-4	BL1	0.6657	0.6657	0.6657	0.6657	0.6657	0.6657
	BL2	—	0.7132	0.7819	0.7745	0.8431	0.8397
	BL3	0.7944	0.7564	0.7506	0.7587	0.7988	0.7993
	TSVM	0.8789	0.8897	0.8942	0.8864	0.8911	0.9001
	ARRLS	0.8654	0.8412	0.8553	0.8631	0.8745	0.8924
	OS-JDA-MR-T-TSK-FC	0.8542	0.8596	0.9241	0.9321	0.9365	0.9387
SC-5	BL1	0.8498	0.8498	0.8498	0.8498	0.8498	0.8498
	BL2	—	0.6349	0.7119	0.7333	0.7425	0.7773
	BL3	0.7751	0.7607	0.7758	0.7677	0.8121	0.8364
	TSVM	0.9024	0.9354	0.9142	0.9321	0.9368	0.9410
	ARRLS	0.8963	0.9224	0.9021	0.9361	0.9556	0.9254
	OS-JDA-MR-T-TSK-FC	0.8654	0.8684	0.9023	0.9234	0.9257	0.9341
SC-6	BL1	0.8267	0.8267	0.8267	0.8267	0.8267	0.8267
	BL2	—	0.6921	0.6723	0.7636	0.8667	0.8283
	BL3	0.7926	0.7743	0.7689	0.7908	0.7946	0.7683
	TSVM	0.8756	0.8632	0.8786	0.8801	0.8698	0.8841
	ARRLS	0.8654	0.8604	0.8552	0.8742	0.8536	0.8774
	OS-JDA-MR-T-TSK-FC	0.8120	0.8763	0.8796	0.8652	0.8605	0.8697

**Table 5:** Classification performance on six scenarios in the WPD feature space.

	***M***	**0**	**4**	**8**	**12**	**16**	**20**
SC-1	BL1	0.9711	0.9711	0.9711	0.9711	0.9711	0.9711
	BL2	—	0.6718	0.9166	0.9142	0.9243	0.9513
	BL3	0.8632	0.7986	0.8542	0.8611	0.8511	0.8442
	TSVM	0.9735	0.9653	0.9842	0.9811	0.9765	0.9647
	ARRLS	0.9632	0.9553	0.8745	0.9567	0.9651	0.9663
	OS-JDA-MR-T-TSK-FC	0.9271	0.9365	0.9654	0.9689	0.9714	0.9736
SC-2	BL1	0.8626	0.8626	0.8626	0.8626	0.8626	0.8626
	BL2	—	0.5873	0.8135	0.8363	0.8627	0.8751
	BL3	0.7895	0.8463	0.8468	0.8532	0.8324	0.8574
	TSVM	0.9021	0.9234	0.9145	0.9310	0.9256	0.9345
	ARRLS	0.8954	0.9321	0.9236	0.9524	0.9125	0.9263
	OS-JDA-MR-T-TSK-FC	0.8852	0.9024	0.9210	0.9253	0.9356	0.9363
SC-3	BL1	0.8388	0.8388	0.8388	0.8388	0.8388	0.8388
	BL2	—	0.8095	0.8067	0.8327	0.8287	0.8865
	BL3	0.7986	0.8023	0.8235	0.8310	0.8352	0.8298
	TSVM	0.8836	0.8896	0.8658	0.8874	0.8697	0.8920
	ARRLS	0.8759	0.8963	0.8741	0.8523	0.8478	0.8623
	OS-JDA-MR-T-TSK-FC	0.7968	0.8541	0.8553	0.8687	0.8723	0.8852
SC-4	BL1	0.9024	0.9024	0.9024	0.9024	0.9024	0.9024
	BL2	—	0.7778	0.9830	0.9818	0.9882	0.9957
	BL3	0.9123	0.9089	0.9189	0.9214	0.9241	0.9298
	TSVM	0.9436	0.9426	0.9463	0.9500	0.9431	0.9498
	ARRLS	0.9355	0.9664	0.9354	0.9632	0.9311	0.9522
	OS-JDA-MR-T-TSK-FC	0.8936	0.9214	0.9386	0.9399	0.9289	0.9400
SC-5	BL1	0.7930	0.7930	0.7930	0.7930	0.7930	0.7930
	BL2	—	0.9047	0.8757	0.8460	0.9454	0.9091
	BL3	0.8826	0.8854	0.8898	0.8754	0.9356	0.9367
	TSVM	0.9241	0.9265	0.9321	0.9222	0.9412	0.9398
	ARRLS	0.9021	0.9214	0.8954	0.8857	0.9145	0.9236
	OS-JDA-MR-T-TSK-FC	0.9311	0.9354	0.9512	0.9568	0.9612	0.9544
SC-6	BL1	0.8029	0.8029	0.8029	0.8029	0.8029	0.8029
	BL2	—	0.5397	0.7627	0.9090	0.8849	0.8879
	BL3	0.8569	0.8601	0.8635	0.8686	0.8720	0.8789
	TSVM	0.9124	0.9154	0.9187	0.9156	0.9189	0.9257
	ARRLS	0.9214	0.9220	0.9201	0.9258	0.9361	0.9123
	OS-JDA-MR-T-TSK-FC	0.8845	0.8942	0.9354	0.9289	0.9298	0.9364

**Table 6:** Classification performance on six scenarios in the STFT feature space.

	***M***	**0**	**4**	**8**	**12**	**16**	**20**
SC-1	BL1	0.8915	0.8915	0.8915	0.8915	0.8915	0.8915
	BL2	—	0.6825	0.7627	0.8400	0.8248	0.8680
	BL3	0.8469	0.8500	0.8598	0.8541	0.8745	0.9021
	TSVM	0.9235	0.9265	0.9211	0.9365	0.9410	0.9389
	ARRLS	0.9123	0.9025	0.9145	0.9452	0.9321	0.9225
	OS-JDA-MR-T-TSK-FC	0.9231	0.9212	0.9536	0.9456	0.9589	0.9610
SC-2	BL1	0.9572	0.9572	0.9572	0.9572	0.9572	0.9572
	BL2	—	0.8412	0.9152	0.8363	0.9215	0.9148
	BL3	0.9356	0.9398	0.9410	0.9369	0.9459	0.9502
	TSVM	0.9578	0.9689	0.9712	0.9754	0.9741	0.9710
	ARRLS	0.9421	0.9532	0.9456	0.9623	0.9456	0.9361
	OS-JDA-MR-T-TSK-FC	0.9241	0.9254	0.9698	0.9789	0.9874	0.9863
SC-3	BL1	0.9452	0.9452	0.9452	0.9452	0.9452	0.9452
	BL2	—	0.8730	0.8983	0.9600	0.9346	0.9148
	BL3	0.9563	0.9541	0.9568	0.9642	0.9687	0.9610
	TSVM	0.9478	0.9620	0.9536	0.9587	0.9641	0.9638
	ARRLS	0.9361	0.9521	0.9357	0.9430	0.9347	0.9637
	OS-JDA-MR-T-TSK-FC	0.9147	0.9689	0.9700	0.9453	0.9432	0.9564
SC-4	BL1	0.9004	0.9004	0.9004	0.9004	0.9004	0.9004
	BL2	—	0.7619	0.8813	0.8363	0.8823	0.9078
	BL3	0.9214	0.9154	0.9354	0.9410	0.9258	0.9320
	TSVM	0.9425	0.9489	0.9631	0.9562	0.9511	0.9468
	ARRLS	0.9364	0.9258	0.9567	0.9412	0.9368	0.9387
	OS-JDA-MR-T-TSK-FC	0.9023	0.9128	0.9587	0.9599	0.9610	0.9632
SC-5	BL1	0.9064	0.9064	0.9064	0.9064	0.9064	0.9064
	BL2	—	0.7778	0.9322	0.8727	0.9424	0.9177
	BL3	0.8921	0.8525	0.8651	0.8621	0.8547	0.8854
	TSVM	0.9257	0.9365	0.9278	0.9421	0.9532	0.9544
	ARRLS	0.9025	0.9236	0.9123	0.9367	0.9458	0.9422
	OS-JDA-MR-T-TSK-FC	0.8789	0.9024	0.9268	0.9541	0.9587	0.9635
SC-6	BL1	0.8766	0.8766	0.8766	0.8766	0.8766	0.8766
	BL2	—	0.6349	0.7288	0.8593	0.8183	0.8539
	BL3	0.8541	0.8423	0.7963	0.8125	0.8236	0.8333
	TSVM	0.9214	0.9325	0.9432	0.9323	0.9654	0.9398
	ARRLS	0.9123	0.9236	0.9347	0.9415	0.9523	0.9225
	OS-JDA-MR-T-TSK-FC	0.8756	0.8974	0.9214	0.9265	0.9421	0.9412

Unlike TSVM that works in a black-box manner, the proposed OS-JDA-MR-T-TSK-FC has high interpretability because 1-TSK-FC is taken as the basic component. Table 7 shows the five trained fuzzy rules (antecedent and consequent parameters) on SC-1 in the KPCA feature space.

- Robustness

**Table 7:** Fuzzy rules trained on SC-1 in the KPCA feature space.

**OS-JDA-MR-T-TSK-FC**			
**Fuzzy rules: If x**_**1**_ **is** ${\text{A}}_{1}^{k}\wedge$***x***_**2**_ **is** ${\text{A}}_{2}^{k}\wedge \ldots \wedge$***x***_***d***_ **is** ${\text{A}}_{d}^{k}$**, then** ${\text{f}}^{k}\left(\text{x}\right)=p_{0}^{k}+p_{1}^{k}x_{1}+\ldots +p_{d}^{k}x_{d},k=1,2,\ldots ,\text{K}$			
SC-1	Rule No.	Antecedent parameters ${\text{c}}^{k}={\left[c_{1}^{k},c_{2}^{k},\ldots ,c_{d}^{k}\right]}^{T},\delta^{k}={\left[\delta_{1}^{k},\delta_{2}^{k},\ldots ,\delta {\;}_{d}^{k}\right]}^{T}$	Consequent parameters ${\text{p}}^{k}={\left[p_{0}^{k},p_{1}^{k},\ldots ,p_{d}^{k}\right]}^{T}$
	1	**c**^1^ = [0.0081, -0.0014, -0.0027, -0.0032, -0.0043, -0.0031], δ^1^ = [0.0023, 0.0055, 0.0036, 0.0041, 0.0021, 0.0028]	**p**^1^ = [0.2531, 0.4321, −0.5123, 025623, 0.2415, −0.0423, 0.0012; 0.3135, 0.5287, 0.4452, −0.5342, 0.2342, −0.9734, −0.3244]^*T*^
	2	**c**^2^ = [0.0055, 0.0031, -0.0023, 0.0022, -0.0098, -0.0021], δ^2^ = [0.0050, 0.0036, 0.0043, 0.0044, 0.0041, 0.0033]	**p**^2^ = [0.1213, −0.5354, 0.5653, −0.1243, 0.3452, 0.0642, 0.0043; 0.0633, −0.6342, 0.1453, 0.3345, −0.0234, 0.0078, −0.0015]^*T*^
	3	**c**^3^ = [0.0498, 0.0411, 0.0014, 0.0056, 0.0016, -0.0028], δ^3^ = [0.0046, 0.0034, 0.0057, 0.0057, 0.0046, 0.0037]	**p**^3^ = [0.2342, −0.8456, −0.6345, −0.0134, −0.0267, 0.0111, −0.0042; −0.0534, 0.0324, 0.0434, 0.0116, 0.0362, −0.0632, 0.0027]^*T*^
	4	**c**^4^ = [0.0673, 0.0432, 0.0014, 0.0057, 0.0014, -0.0033], δ^4^ = [0.0041, 0.0032, 0.0032, 0.0011, 0.0034, 0.0015]	**p**^4^ = [0.0454, −0.4345, −0.2563, −0.0412, 0.0345, 0.0163, 0.0423; 0.0123, −0.0532, 0.1634, 0.2134, −0.0745, 0.0122, 0.0011]^*T*^
	5	**c**^5^ = [0.0042, 0.0098, 0.0015, 0.0034, 0.0047, -0.0011], δ^5^ = [0.0047, 0.0032, 0.0044, 0.0076, 0.0034, 0.0043]	**p**^5^ = [0.0177, 0.0134, 0.0214, 0.0034, −0.0045, 0.0023, −0.0013; 0.0034, 0.0053, −0.0123, 0.0054, 0.0053, 0.0016, 0.0014]^*T*^

**Figure 7:**
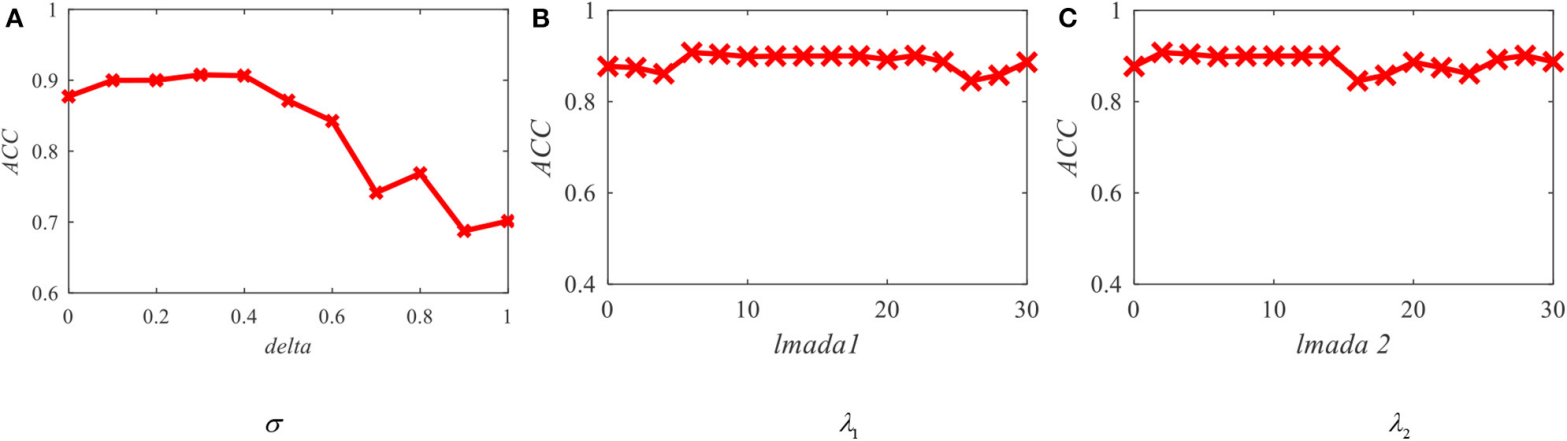
Average accuracy of OS-JDA-MR-T-TSK-FC in the KPCA feature space with different parameters. **(A)** Robustness w.r.t delta; **(B)** robustness w.r.t lmada 1; **(C)** robustness w.r.t lmada 2.

From the objective function of OS-JDA-MR-T-TSK-FC, we see that there are three parameters, that is, ω_*t*_ (σ), λ_1_, and λ_2_ that should be fixed before a classification task. So, we should consider the robustness OS-JDA-MR-T-TSK-FC to them. The sensitivity analysis results are shown in Figure 7.

## Discussions

We observe from Table 3 that the proposed OS-JDA-MR-T-TSK-FC wins the best average performance across the six transfer scenarios in all feature spaces when the number of specific-subject objects is more than 4. Especially compared with the three baselines, the advantages are more obvious.

Moreover, the classification results in Tables 4–6 also exhibit the following four characteristics:

- BL1 does not use the specific-subject objects, so its accuracy is independent on *M*, whereas the other four classifiers depend on *M*, and it is intuitive that they gradually perform better than BL1 with the increasing of *M*.
- BL2 is only trained by the subject-specific objects. Therefore, BL2 becomes unusable when *M* is set to 0. But BL1, BL3, TSVM, and OS-JDA-MR-T-TSK-FC can work because, except subject-specific objects, they also leverage training objects from the source domains. Compared with other algorithms, when *M* is too small, BL2 performs so badly because it cannot get enough training patterns from subject-specific objects.
- When *M* is set to 0, TSVM always achieves the best performance. With the subject-specific objects gradually added into the training set, OS-JDA-MR-T-TSK-FC soon performs better than TSVM, which indicates that significant differences exist among the domains. Hence, a domain-dependent classifier, for example, TSVM is not very expected in our online transfer scenarios.
- When one batch (four subject-specific objects are taken as a batch in our experiments) or at most two batches of subject-specific objects are added into the training set, the classification performance of OS-JDA-MR-T-TSK-FC becomes stable. That is to say, the number of subject-specific objects OS-JDA-MR-T-TSK-FC needs is very small. So, OS-JDA-MR-T-TSK-FC meets the practical requirements because subject-specific objects are very few in real-world applications.

In addition to classification performance, interpretability is also a main characteristic of the proposed OS-JDA-MR-T-TSK-FC. From Table 7, we see that it generates five interpretable fuzzy rules on SC-1 in the KPCA feature space. Each feature in a fuzzy rule can be interpreted as the energy of an EEG signal band, and each fuzzy membership function is endowed with a linguistic description. For example, “*x*_1_ is $A_{1}^{k}$” in the antecedent of a fuzzy rule can be interpreted as “the energy of an EEG band is a litter high,” where the term “a little high” can be replaced by others such as “a litter low,” “medium,” or “high.” In this way, suppose I am an expert from the field of EEG signal analysis, I assign five kinds of linguistic descriptions to each fuzzy membership function, that is, “low,” “a little low,” “medium,” “a little high,” and “high.” Therefore, for the first fuzzy rule in Table 7, it can be interpreted as follows:

*If the energy of an EEG signal band (band 1) is “high,” and the energy of an EEG signal band (band 2) is “a little low,” and the energy of an EEG signal band (band 3) is “low,” and the energy of an EEG signal band (band 4) is “low,” and the energy of an EEG signal band (band 5) is “low,” and the energy of an EEG signal band (band 6) is “low,”* ***then*** *the consequent of the first fuzzy rule can be expressed as:*

$f^{1}\left(\text{x}\right)=0.2531+0.4321x_{1}-0.5123x_{2}+0.2562x_{3}+0.2415x_{4}-0.0423x_{5}+0.0012x_{6}+0.3153$−0.5278*x*_1_+0.4452*x*_2_−0.5342*x*_3_+0.2342*x*_4_−0.9734*x*_5_−0.3244*x*_6_.

From Figure 6, we observe that O-T-TSK-FC is robust to σ in the range of [0.1, 0.4], to λ_1_ in the range of (Geng et al., 2012; Jiang et al., 2017c), and to λ_2_ in the range of (Ghosh-Dastidar et al., 2008; Mallapragada et al., 2009), respectively.

## Conclusions

In this study, we propose a seizure classification model OS-JDA-MR-T-TSK-FC using an online selective transfer TSK fuzzy classifier with a joint distribution adaption and manifold regularization. We use epilepsy EEG signals provided by the University of Bonn as the original data and construct six transfer scenarios in three kinds of feature spaces to demonstrate the promising performance of OS-JDA-MR-T-TSK-FC. We also generate four baselines and introduce a transfer SVM model for fair comparison. The experimental results show that OS-JDA-MR-T-TSK-FC performs better than baselines and the introduced two transfer models. However, in this study, we only consider how to select the source domains. Recent studies show that dynamically selecting useful samples from the source domain can effectively induce the learning on the target domain. Therefore, in our future work, we will try to develop a mechanism, for example, classification error consensus to select most useful samples from the source domain.

## Data Availability Statement

The original EEG data are available in http://www.meb.unibonn.de/epileptologie/science/physik/eegdata.html.

## Author Contributions

YZ designed the whole algorithm and experiments. ZZ, HB, and WL contributed on code implementation, and LW gave some suggestions to the writing.

## Conflict of Interest

The authors declare that the research was conducted in the absence of any commercial or financial relationships that could be construed as a potential conflict of interest.

## Footnotes

^1^http://www.meb.unibonn.de/epileptologie/science/physik/eegdata.html.
